# In vivo imaging of tumour xenografts with an antibody targeting the potassium channel K_v_10.1

**DOI:** 10.1007/s00249-016-1152-z

**Published:** 2016-07-21

**Authors:** Joanna Napp, Luis A. Pardo, Franziska Hartung, Lutz F. Tietze, Walter Stühmer, Frauke Alves

**Affiliations:** 1 Department of Molecular Biology of Neuronal Signals, Max Planck Institute of Experimental Medicine, Hermann-Rein-Straße 3, 37075 Göttingen, Germany; 2 AG Oncophysiology, Max Planck Institute of Experimental Medicine, Hermann-Rein-Straße 3, 37075 Göttingen, Germany; 3 Institute of Interventional and Diagnostic Radiology, University Medical Center Göttingen, Robert-Koch-Str. 40, 37075 Göttingen, Germany; 4 Department of Haematology and Medical Oncology, University Medical Center Göttingen, Robert-Koch-Str. 40, 37075 Göttingen, Germany; 5 Institute of Organic and Biomolecular Chemistry, University Göttingen, Tammannstr. 2, 37077 Göttingen, Germany

**Keywords:** Antibody targeting K_v_10.1, Ion channel K_v_10.1, Non-invasive near infrared fluorescence imaging, Preclinical assessment of enzymatic activity, Novel therapeutic concepts, Oncology

## Abstract

The K_v_10.1 (Eag1) voltage-gated potassium channel represents a promising molecular target for novel cancer therapies or diagnostic purposes. Physiologically, it is only expressed in the brain, but it was found overexpressed in more than 70 % of tumours of diverse origin. Furthermore, as a plasma membrane protein, it is easily accessible to extracellular interventions. In this study we analysed the feasibility of the anti-K_v_10.1 monoclonal antibody mAb62 to target tumour cells in vitro and in vivo and to deliver therapeutics to the tumour. Using time-domain near infrared fluorescence (NIRF) imaging in a subcutaneous MDA-MB-435S tumour model in nude mice, we showed that mAb62-Cy5.5 specifically accumulates at the tumour for at least 1 week in vivo with a maximum intensity at 48 h. Blocking experiments with an excess of unlabelled mAb62 and application of the free Cy5.5 fluorophore demonstrate specific binding to the tumour. Ex vivo NIRF imaging of whole tumours as well as NIRF imaging and microscopy of tumour slices confirmed the accumulation of the mAb62-Cy5.5 in tumours but not in brain tissue. Moreover, mAb62 was conjugated to the prodrug-activating enzyme β-D-galactosidase (β-gal; mAb62-β-gal). The β-gal activity of the mAb62-β-gal conjugate was analysed in vitro on K_v_10.1-expressing MDA-MB-435S cells in comparison to control AsPC-1 cells. We show that the mAb62-β-gal conjugate possesses high β-gal activity when bound to K_v_10.1-expressing MDA-MB-435S cells. Moreover, using the β-gal activatable NIRF probe DDAOG, we detected mAb62-β-gal activity in vivo over the tumour area. In summary, we could show that the anti-K_v_10.1 antibody is a promising tool for the development of novel concepts of targeted cancer therapy.

## Introduction

Despite intense medical and research efforts during recent decades, cancer is still far from being curable and remains a leading cause of death worldwide. Consequently, there is still a substantial need to improve the existing and to develop alternative strategies to detect and to treat cancer.

Several trends can be observed in cancer research, each of extreme importance for both the improvement of current tumour diagnostics and the development of novel therapeutic interventions: (1) basic research for better understanding of molecular mechanisms underlying cancer biology, (2) identification and extensive characterisation of novel molecular targets and/or tools and (3) development of increasingly sophisticated therapy concepts.

Among many other tumour-specific targets, an increased interest has focused on ion channels, as evidence relates them to the pathogenesis of malignancies (Lang and Stournaras 2014; Pardo and Stuhmer 2014; Arcangeli and Becchetti 2015). Ion channels are transmembrane proteins predominantly expressed on the cell surface, accessible to the extracellular space and therefore to external interventions, facilitating their use in diagnosis and therapy (Pardo and Stuhmer 2014). The ether-à-go–go 1 (K_v_10.1; Eag1) voltage-gated potassium channel is a promising target. In contrast to its restricted distribution in normal healthy tissue, K_v_10.1 is significantly overexpressed in many tumour cell lines and in a variety of solid tumours from different histological origins such as breast, colon or cervix (Hemmerlein et al. 2006; Mello de Queiroz et al. 2006; Ding et al. 2007a, b). Furthermore, cells aberrantly overexpressing K_v_10.1 acquire phenotypical characteristics of malignancy and induce strongly aggressive tumour growth in immunodeficient mice (Pardo et al. 1999).

In fact, the efficacy of K_v_10.1-targeting antibodies and blockers on inhibition/reduction of tumour growth has already been described although the exact mechanisms remain unclear. Knockdown or blocking of K_v_10.1 with siRNA or a monoclonal antibody selectively inhibiting K_v_10.1-mediated potassium currents reduced the proliferation of cancer cell lines and tumour growth in in vivo models (Weber et al. 2006; Gomez-Varela et al. 2007; Downie et al. 2008). K_v_10.1 is not only expressed in the primary tumours, but also in brain metastases, where it might contribute to tumour progression, because patients with brain metastases and moderate K_v_10.1 expression showed improved survival when treated with different K_v_10.1-blocking antidepressants (Martinez et al. 2015) compared with those treated with other antidepressant drugs. Moreover, a fusion protein of single-chain K_v_10.1-targeting antibody and tumour necrosis factor-related apoptosis inducing ligand (TRAIL) were shown to not only specifically induce apoptosis of tumour cells, but also to sensitise them for chemotherapeutic agents (Hartung et al. 2011; Hartung and Pardo 2016).

Although K_v_10.1-targeting antibodies have already been suggested for tumour imaging (Mello de Queiroz et al. 2006), none of them has been systematically characterised for an in vivo application. Here we present an extensive characterisation of the K_v_10.1-targeting monoclonal antibody mAb62 (Hemmerlein et al. 2006) in vitro and in vivo using near infrared (NIR) imaging in mouse tumour models in order to evaluate its applicability for diagnostic and therapeutic purposes.

## Materials and methods

### Cell culture

Human melanoma MDA-MB-435S and human pancreatic carcinoma AsPC-1 cell lines were obtained from ATCC (Rockville MD). Cells were cultured in RPMI 1640 medium with GlutaMAX supplemented with 10 % fetal calf serum (FCS; Invitrogen).

### Real-time PCR

Total RNA was extracted with the RNeasy mini kit (Qiagen) following the manufacturer’s recommendations; 5 µg of total RNA was used for cDNA synthesis with SuperScript (Invitrogen).

Real-time PCR was performed with 100 ng cDNA in a LightCycler 480 (Roche) as previously described (Hartung et al. 2011). Human transferrin receptor was used as a reference. The specific mRNA content was determined using the LightCycler 480 software (Roche). All measurements were performed in triplicate.

### Antibody conjugates

The monoclonal mouse antibody mAb62 (IgG κ2b) generated in our group (Gomez-Varela et al. 2007; Ramos Gomes et al. 2015) was labelled with Cy5.5 (Cy5.5 maleimide monoreactive dye; Amersham/GE Healthcare Life Sciences) using a standard protocol, resulting in mAb62-Cy5.5 (dye/protein ratio of ~0.7).

The coupling of mAb62 with β-D-galactosidase (G4155; Sigma) resulting in mAb62-β-gal was performed by Squarix GmbH (Marl, Germany).

### Antibody-binding assay

The 96-well plates were coated overnight at 4 °C with 500 ng of h1x antigen (Hemmerlein et al. 2006) in 100 µl Tris-buffered saline (TBS). After blocking with 200 µl 3 % bovine serum albumin (BSA) in TBS for 1 h at RT, 1 µg of mAb62-Cy5.5 antibody in 100 µl TBS was added to each well and plates were incubated at RT for 2 h. For competitive binding studies, increasing amounts of unlabelled mAb62 (0.04–4 µg/well) were added to the wells and incubated together with 1 µg of mAb62-Cy5.5. Subsequently, the plates were washed 3 × 10 min with 0.05 % Tween 20 in TBS, and 100 µl TBS buffer was added to each well. The plates were scanned in an Odyssey Infrared Imager (LI-COR Biosciences) using 700 nm excitation. To quantify the binding, a circular ROI of identical size was drawn over the centre of each well and the average fluorescence intensity over the area was measured in arbitrary units (a.u.). The experiment was performed in duplicate.

### Immunofluorescence staining and microscopy

Prior to the experiments 5 × 10^5^ AsPC-1 or MDA-MB-435S cells per well were grown overnight on coverslips in 24-well plates.

For immunofluorescence staining, cells were fixed for 10 min with ice-cold 4 % paraformaldehyde (PFA), permeabilised for 10 min with 0.5 % Triton X-100 in phosphate-buffered saline (PBS) and washed three times with PBS. Then cells were blocked for 10 min with 10 % BSA in PBS and incubated with mAb62 (5 µg in 200 µl of 10 % BSA) in PBS for 2 h at room temperature, washed three times each for 10 min with 0.1 % Tween 20 in PBS and stained with anti-mouse Alexa Fluor 488 antibody (Invitrogen; Molecular Probes) for 30 min at room temperature. Finally, cells were washed three times for 10 min with 0.1 % Tween 20 in PBS, rinsed with PBS and mounted with ProLong Gold Antifade Reagent with DAPI (Invitrogen).

For staining of living cells, the medium was replaced by fresh culture medium containing 10 µg/ml mAb62-Cy5.5 and cells were left at 37 °C for another 3 h. Subsequently, cells were washed with PBS, fixed in 4 % PFA/PBS and mounted with ProLong Gold Antifade Reagent with DAPI (Invitrogen).

Cells were imaged with an Axiovert 200 M fluorescence microscope (Carl Zeiss) equipped with a NIR-sensitive ORCA-ER digital camera (Hamamatsu) with the following filter settings: 365 ± 12.5 nm excitation and a 445 ± 25 nm emission filter for DAPI, 450–490 nm excitation and 515–565 nm emission filter for Alexa Fluor 488 and 640 ± 15 nm excitation and 690 ± 25 nm emission filter for Alexa-Fluor 647. Image generation and processing were performed with the AxioVision Rel.4.6 and ImageJ software (Collins 2007), respectively.

### In vitro imaging of β-gal activity

1 × 10^5^ of AsPC-1 or MDA-MB-435S cells was plated in 96-well plates and allowed to attach for 24 h. The medium was then replaced with 50 µl of staining buffer (2 % FCS, 0.1 % NaN_3_ in PBS) containing 2.5 µg of mAb62-β-gal and the cells were incubated with the conjugate for 30 min on ice. After four washing steps, each by immersing the entire plate for 1 min into a tray containing 1 l of washing buffer (130 mM NaCl, 10 mM HEPES pH 7.4, 5 mM KCl, 1 mM CaCl_2_ and 1 % FCS), 100 µl of freshly prepared substrate solution containing 0.1 % NaN_3_, 1 % BSA, 2 mM MgCl_2_ and 2 mg/ml chlorophenol red-β-d-galactopyranoside (CPRG; Sigma) in PBS was added to each well and the increase of absorbance at 570 nm over time was measured immediately in a Wallac 1420 Victor2 Microplate Reader (Perkin Elmer). Five wells were analysed per measurement.

### Animal models

Female athymic nude NMRI-Fox1^nu/nu^ mice (Winkelmann) were used for the in vivo imaging experiments. Animals were handled according to German ethics regulations for animal experimentation and all protocols were approved by the administration of Lower Saxony, Germany.

Mice were anaesthetised with 1.8–2.0 % inhaled isoflurane and either 1 × 10^7^ MDA-MB-435S or 1 × 10^6^ AsPC1 cells suspended in 100 µl of PBS were subcutaneously transplanted into the right flank of the mice. Mice were inspected twice weekly for tumour development, loss of body weight and general condition. All animals tolerated the procedure well. At the end of the experiment animals were euthanized by isoflurane overdose and cervical dislocation.

### NIRF imaging

To reduce the fluorescence background, mice received chlorophyll-reduced GLP Nafag 890 food (Provimi Kliba AG) for ~1 week before NIRF imaging. All in vivo analyses were preceded by native scans.

Mice were anaesthetised with 1.8–2.0 % inhaled isoflurane and injected intravenously (i.v.) via the tail vein with either 25 µg of mAb62-Cy5.5, 25 µg of mAb62-Cy5.5 together with 50 µg of unlabelled mAb62 or 1.2 µg of Cy5.5 (Amersham) in 150 µl NaCl 0.9 %. Animals were scanned with the Optix MX2 (ART) time-domain imaging system in vivo at the indicated times. At the end of experiments, mice were sacrificed and the excised organs were imaged ex vivo.

Cy5.5 fluorescence was obtained with an excitation at 670 nm in combination with a 700-nm long-pass emission filter. Scans were performed with a 1.5 mm (whole-body scans and organ scans) or 1.0 mm (region of interest, ROI) raster and a point integration time of 0.7 s. Data were analysed with OptiView software (ART).

Unspecific signals were identified by lifetime measurement. Lifetimes (LTs) that deviated from measured values of the ligand-dye conjugates in vitro were considered as background, which was removed from intensity images by setting lifetime gates (Napp et al. 2010).

For in vivo measurement of β-gal activity (Tung et al. 2004) 25 µg of mAb62-β-gal was applied i.v. to tumour-bearing mice. After ~24 h, isoflurane-anaesthetised animals received i.v. 5 mg of DDAOG [(9H-(1,3-dichloro-9,9-dimethylacridin-2-One-7-yl) β-d-galactopyranoside), Molecular Probes/Thermofisher] in 150 µl of 0.9 % NaCl/DMSO (50/50). Mice were repetitively scanned over ~3.5 h with the Optix MX2 directly after injection using a 635 nm excitation laser and a 670 ± 20 nm emission filter, 1.0–1.5 mm raster and a point integration time of 0.7 s.

### Imaging on tissue slices

Tumour and brain tissue from mice injected with mAb62-Cy5.5 24 h prior to the dissection were fixed over night with 4 % PFA and cut on a vibratome (Leica VT1000 S) into 100 µm-thick sections. The tissue sections were then mounted onto microscope slides and imaged with the flatbed scanner Odyssey NIRF imager (LI-COR Biosciences) using 700 nm settings.

### Statistics

The significance was analysed by two-tailed unpaired Student’s *t* test using the PAST programme (Hammer et al. 2001). Statistical significance was defined as *p* ≤ 0.05.

## Results

### Binding of the anti-K_v_10.1 antibody mAb62-Cy5.5 to K_v_10.1 in vitro

The mouse monoclonal antibody (IgG κ2b) mAb62 was obtained by immunisation using a fusion protein h1x that contains the pore-forming loop and the tetramerisation domain of K_v_10.1 (Hemmerlein et al. 2006; Gomez-Varela et al. 2007). Antibody specificity to K_v_10.1 was determined by surface plasmon resonance against several other channels and on tissues from knockout mice. In vitro evaluation of mAb62 for efficient targeting and monitoring of tumours was performed with the MDA-MB-435S cell line, which is known to express K_v_10.1 and which was used by our group in previous in vivo studies (Gomez-Varela et al. 2007; Downie et al. 2008; Hartung and Pardo 2016). AsPC1 cells were chosen as a control and the K_v_10.1 expression levels were verified in both cell lines by real-time PCR.

**Fig. 1:**
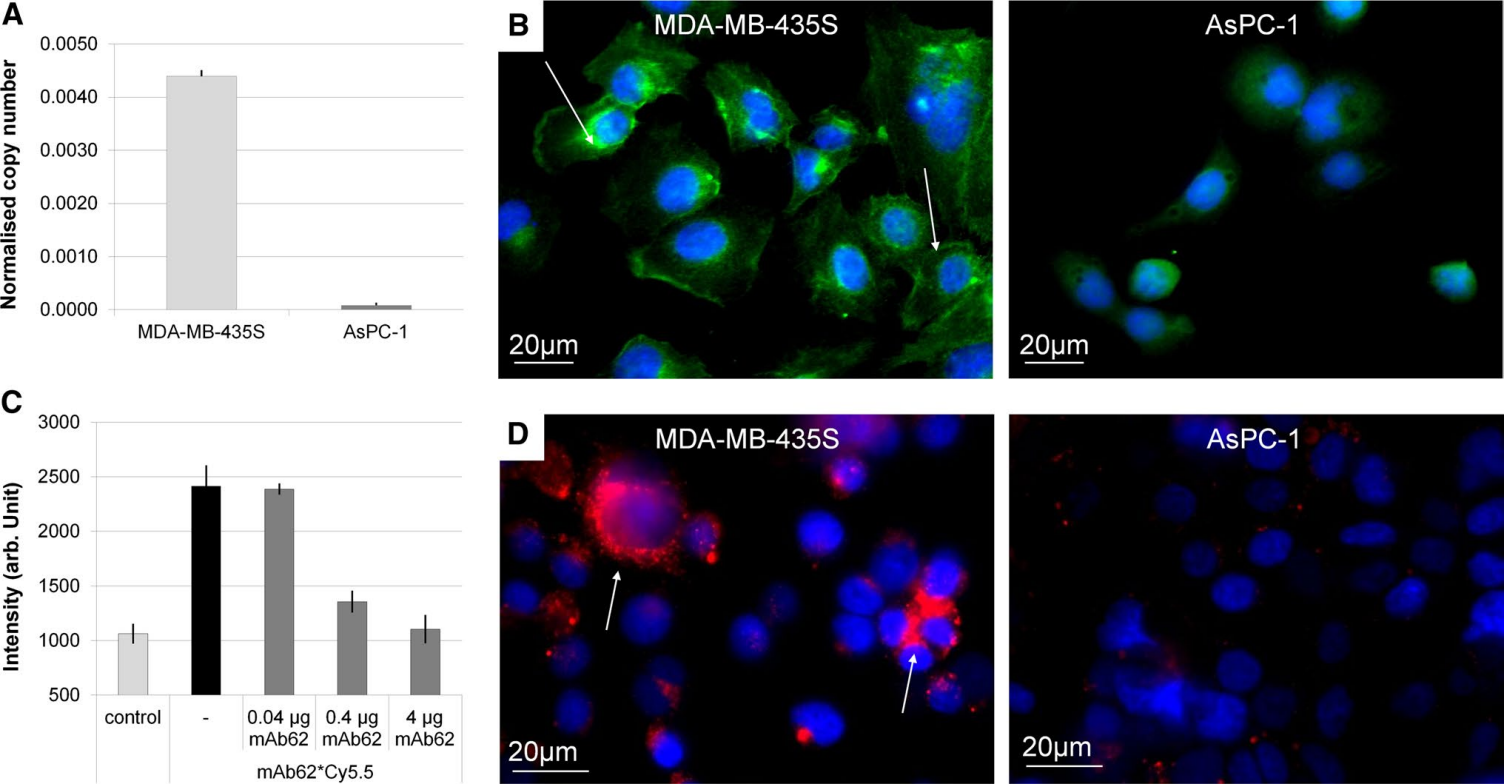
In vitro analysis of mAb62-Cy5.5 binding specificity to K_v_10.1-expressing cells. **a** Real-time PCR shows high K_v_10.1 expression in MDA-MB-435S cells in comparison to the AsPC-1 cells. **b** Staining of fixed MDA-MB-435S (*left*) and AsPC-1 (*right*) cells with mAb62, followed by anti-mouse Alexa 488-labeled secondary antibody is shown. Specific binding of the mAb62 could only be detected on K_v_10.1-expressing MDA-MB-435S cells (*green*; *arrows*), but not on the AsPC-1 cells. **c** Fluorescence based immunoassay shows that mAb62-Cy5.5 specifically binds to the K_v_10.1 epitope h1x. While high average fluorescence intensities can be detected upon incubation of a h1x-coated plate with mAb62-Cy5.5, binding can be reduced by co-incubation with an excess of unlabelled mAb62 in a concentration-dependent manner. Fluorescence intensity is shown in arbitrary units (a.u.). The experiment was performed in duplicate. **d** Immunofluorescence staining of live MDA-MB-435S and AsPC-1 cells with mAb62-Cy5.5 demonstrates strong mAb62-Cy5.5-derived fluorescence (*red*; *arrows*) only in the K_v_10.1-expressing MDA-MB-435S cells. Nuclei are shown in *blue*. *Bars* 20 µm

As shown in Fig. 1a high levels of K_v_10.1 expression were detected in the MDA-MB-435S cells, whereas no K_v_10.1 expression was found in the AsPC-1 cells.

Furthermore, both cell lines were grown on glass coverslips and stained with mAb62, followed by the detection with an anti-mouse secondary antibody labelled with Alexa Fluor 488. As expected, a clear staining was detected only on K_v_10.1 high-expressing MDA-MB-435S but not on AsPC1 cells (Fig. 1b).

For in vivo imaging, the antibody was labelled with Cy5.5, resulting in mAb62-Cy5.5. Binding of the conjugate to the h1x antigen was analysed in an immunoassay, in which the fluorescence intensity upon binding of the Cy5.5-labelled antibody to an h1x antigen-coated plate was measured in the Odyssey NIRF imaging system. As shown in Fig. 1c, mAb62-Cy5.5 specifically binds to the K_v_10.1 epitope in vitro, demonstrated by high average fluorescence intensity. Moreover, upon addition of increasing amounts of free mAb62 (0.04, 0.4 and 4 µg), binding of mAb62-Cy5.5 to the h1x epitope was reduced in a concentration-dependent manner. While the lowest concentration of 0.04 µg showed no effect on the average fluorescence intensity, the binding to h1x could be almost completely blocked with 4 µg of unlabelled mAb62.

Further, we analysed whether mAb62-Cy5.5 conjugate specifically binds to K_v_10.1-expressing cells. For this purpose, living cells were incubated with mAb62-Cy5.5 for 3 h in cell culture medium. As shown in Fig. 1d, binding and uptake were observed only in K_v_10.1-expressing MDA-MB-435S cells, whereas no mAb62-Cy5.5-derived fluorescence was detected in the control AsPC-1 cells that showed no K_v_10.1 expression in real-time PCR. In MDA-MB-435S cells the most intense fluorescence signals were detected as foci, which probably correspond to the internalised probe.

### In vivo binding of the mAb62-Cy5.5 to K_v_10.1-expressing tumours

**Fig. 2:**
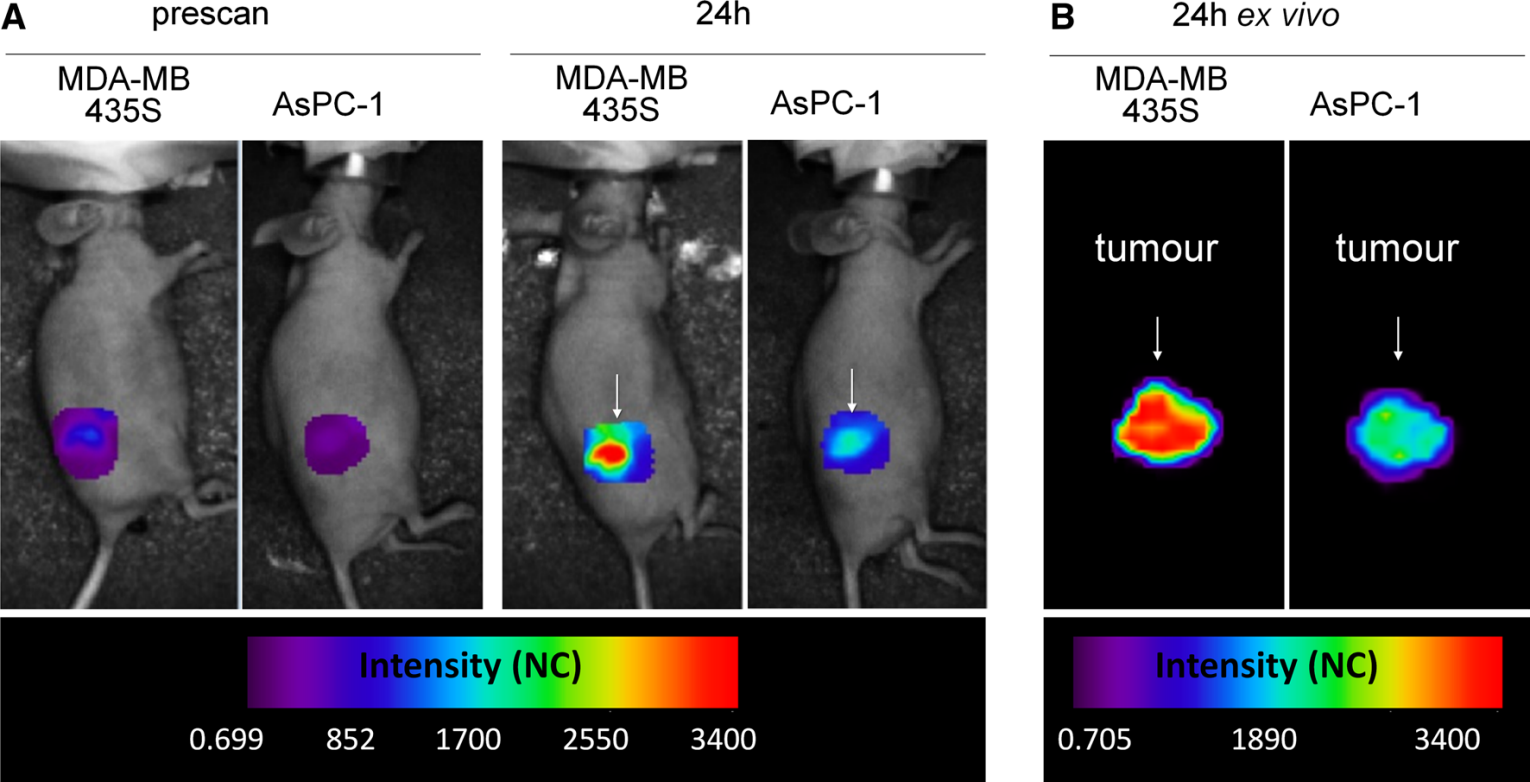
Binding of mAb62-Cy5.5 to K_v_10.1-expressing tumours. Representative **a** in vivo and **b** ex vivo scans of MDA-MB-435S and AsPC-1 subcutaneous tumours 24 h after i.v. injection of mAb62-Cy5.5. High fluorescence intensity was observed over the K_v_10.1-expressing MDA-MB-435S tumour (*arrow*), whereas the control AsPC1 tumour (*arrow*) showed much lower fluorescence in both in vivo and ex vivo scans. Intensity is depicted in normalised counts (NC)

In order to characterise the capability of mAb62 for in vivo tumour imaging and/or targeting, MDA-MB-435S and AsPC-1 cells were subcutaneously transplanted into nude mice. After the tumours reached volumes of ~200 mm^3^, mice were injected with 25 µg of mAb62-Cy5.5 through the tail vein and imaged 24 h later. As shown in Fig. 2a, a strong and specific accumulation of the fluorescent probe to the K_v_10.1-expressing MDA-MB-435S tumours was detected by Optix MX2 in vivo. Interestingly, AsPC-1 tumour-bearing mice also showed an increase of the fluorescence intensity over the tumour area after probe injection, although the signals were much weaker. This fluorescence can be explained by the presence of the probe in the circulating blood and/or by the unspecific accumulation of the antibody in the tumour due to leaky vessels. However, some upregulation of K_v_10.1 expression within the AsPC-1 tumour tissue cannot be excluded.

Twenty-four hours after probe injection mice were killed and the excised tumours were scanned ex vivo (Fig. 2b). As expected, the fluorescence intensity detected over the K_v_10.1-expressing MDA-MB-435S tumour at 24 h was much higher than the one detected over the control AsPC1 tumour.

**Fig. 3:**
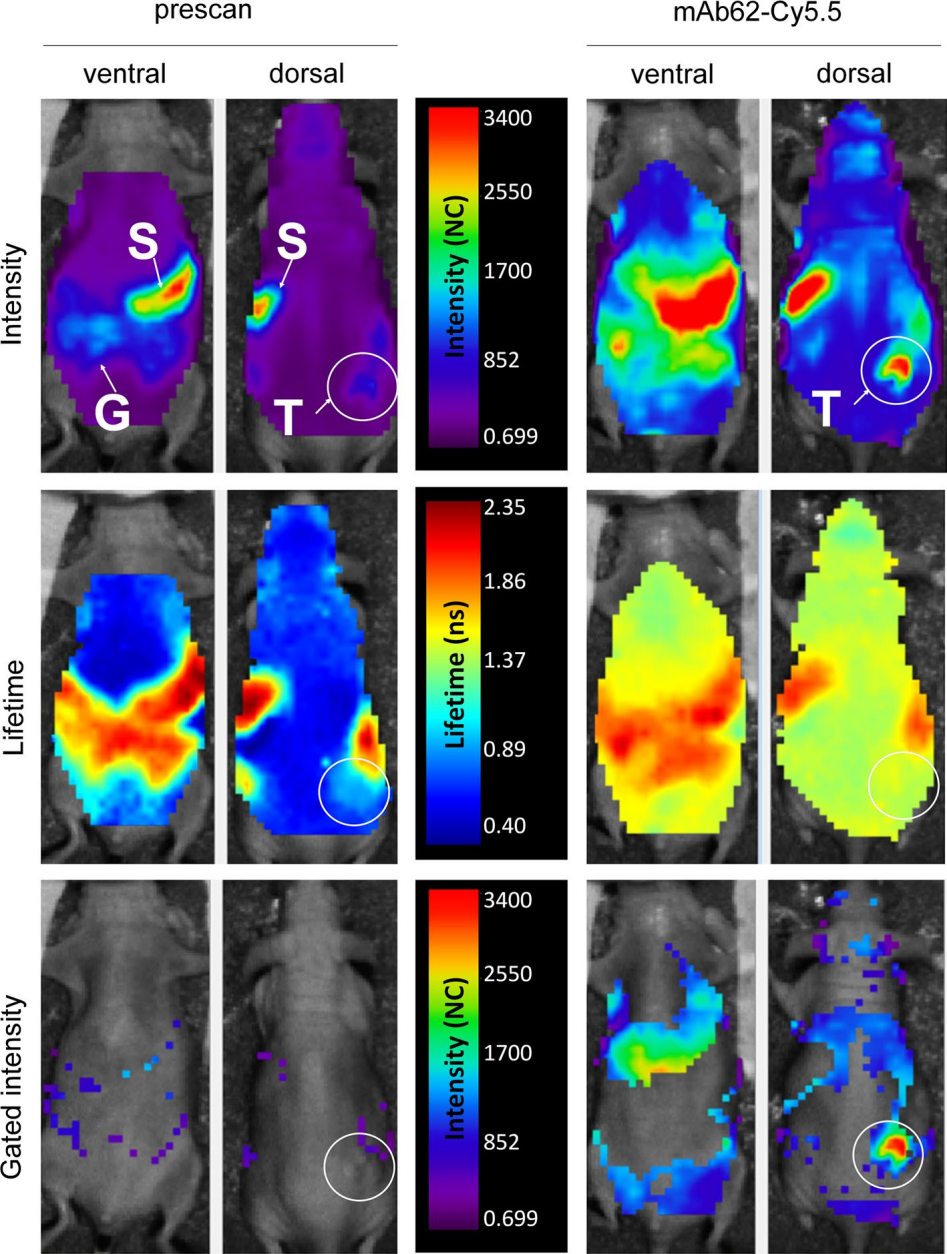
In vivo characterisation of mAb62-Cy5.5-derived fluorescence. Representative intensity and lifetime maps of an MDA-MB-435S tumour-bearing mouse are shown before (*left*; *prescan*) and 24 h after i.v. injection of mAb62-Cy5.5, scanned ventrally and dorsally in vivo with the Optix MX2. *Upper panel* shows fluorescence intensity scans with a low background autofluorescence and signals detected over the stomach (*S*) and gastrointestinal tract (*G*). Strong fluorescence over the K_v_10.1-expressing tumour (*T*) as well as an increase in the overall background was measured 24 h after application of mAb62-Cy5.5. Lifetime (*middle panel*) of the background fluorescence was 0.6–0.8 ns in the prescans and increased to 1.4–1.6 ns after application of the probe, whereas the fluorescence lifetime over the gastrointestinal tract remained the same (1.9–2.3 ns). The *lower panel* shows fluorescence intensity maps after setting the lifetime gate to 1.50–1.65 ns. Note that not only the high fluorescence intensity over the tumour area but also some of the fluorescence over the whole body remained after gating. Fluorescence is shown in normalised counts (NC)

Next, we characterised the binding and distribution pattern of mAb62-Cy5.5 in MDA-MB-435S tumour-bearing mice. Representative data for ventral and dorsal scans obtained at 24 h after probe administration are shown in Fig. 3. In prescans performed before probe administration, a homogeneous autofluorescence background over the whole body was detected as well as a stronger background over the stomach (S) and gastrointestinal tract (G) (Fig. 3; upper panels). As shown in the middle panels of Fig. 3, autofluorescence lifetimes over the whole body were much shorter (0.6–0.8 ns) than the ones determined over the gastrointestinal tract (1.9–2.3 ns). In our experience the fluorescence observed over the stomach and intestinal tract probably derives from food, despite feeding the animals with a chlorophyll-depleted diet (data not shown). The background autofluorescence over the subcutaneous tumours (T; white circle) had slightly higher intensity and a longer lifetime (0.9 ns) than whole-body background autofluorescence levels.

Twenty-four hours after application of mAb62-Cy5.5 strong fluorescence over the tumour could be detected in whole-body scans. Moreover, the fluorescence intensity over the whole body increased, including the area over the stomach and liver as well as the area over the head, visible on the dorsal scans. The lifetime of 1.6 ns measured over the tumour was comparable to the probe’s lifetime measured in vitro (1.5 ns; data not shown) and confirms the specificity of the signal detected in vivo. The lifetime of the fluorescence measured over the background was longer (1.4–1.6 ns) in comparison to that of the prescans (Fig. 3 middle panel), suggesting that the higher background was most likely because of the presence of the circulating mAb62-Cy5.5 probe. The average fluorescence lifetime over the gastrointestinal tract did not change when compared to the prescans.

In order to select the specific probe-derived fluorescence over the tumour, we applied a lifetime gate of 1.50–1.65 ns to the raw scan data. As shown in the lower panel of Fig. 3, we removed most of the background fluorescence from the prescans. As expected, the strong probe-derived fluorescence over the tumour area remained on the gated scans. Moreover, even after gating, fluorescence was still observed over the whole body, probably deriving from the free circulating probe.

**Fig. 4:**
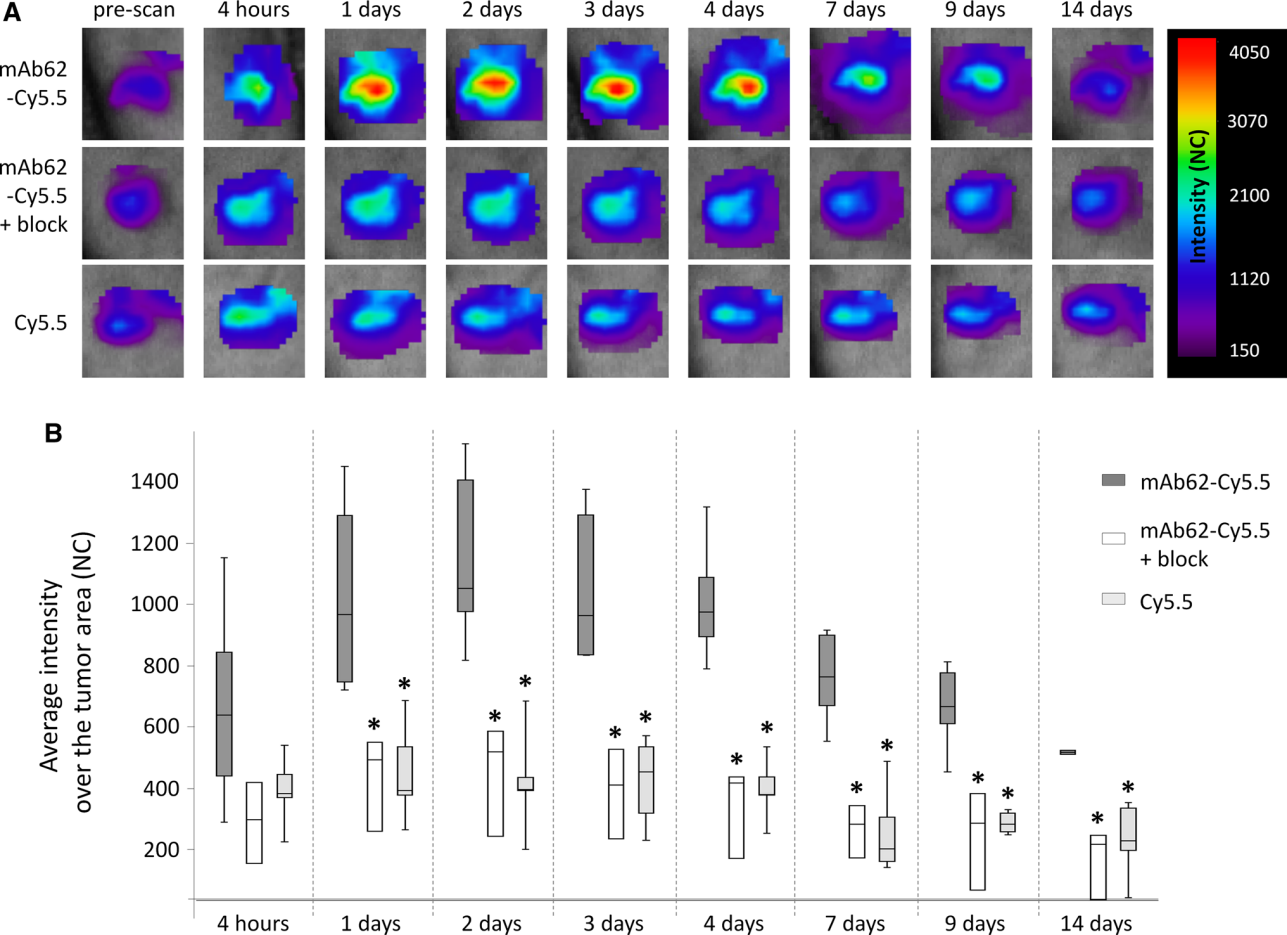
In vivo time-domain fluorescence imaging of subcutaneous K_v_10.1-expressing MDA-MB-435S tumours in mice injected with mAb62-Cy5.5 **a** Representative series of scans over the tumour areas acquired at different time points after injection of 25 µg of mAb62-Cy5.5 (*n* = 8 for 1 day, 2 days and 7 days; *n* = 6 for 4 h, 3 days and 4 days; *n* = 5 for 9 days and *n* = 3 for 14 days), of 25 µg of mAb62-Cy5.5 together with unlabelled mAb62 (*n* = 3) and of 1.2 µg of Cy5.5 (*n* = 5) are shown. **b** *Box* plot showing significantly higher average fluorescence intensities (NC) measured over time over the MDA-MB-435S tumours of mice injected with 25 µg mAb62-Cy5.5 compared to the tumours of mice injected with 25 µg of the mAb62-Cy5.5 together with twofold excess (50 µg) of mAb62 (blocking experiment) or with 1.2 µg of free Cy5.5 fluorophore (*p < 0.05)

In order to test the specificity of the mAb62 in vivo in more detail, either 25 µg of mAb62-Cy5.5, 25 µg of mAb62-Cy5.5 together with a twofold excess (50 µg) of mAb62 (blocking experiment) or 1.2 µg of free Cy5.5 fluorophore was injected into MDA-MB-435S tumour-bearing mice. Time-domain fluorescence imaging of the tumour areas was performed over time, before (prescan), 4 h after as well as 1, 2, 3, 4, 7, 9 and 14 days after probe application. As shown in the representative images of the tumour areas in Fig. 4a, mAb62-Cy5.5-derived fluorescence was detected at the tumour site for at least 1 week with a maximum intensity measured 2 days post injection. Blocking experiments with a twofold excess of unlabelled mAb62 as well as application of the free Cy5.5 fluorophore (Fig. 4b) resulted in significantly lower fluorescence intensities at all time points between 1 and 14 days after injection and therefore confirmed the binding specificity of mAb62-Cy5.5 to K_v_10.1-expressing MDA-MB-435S tumours.

**Fig. 5:**
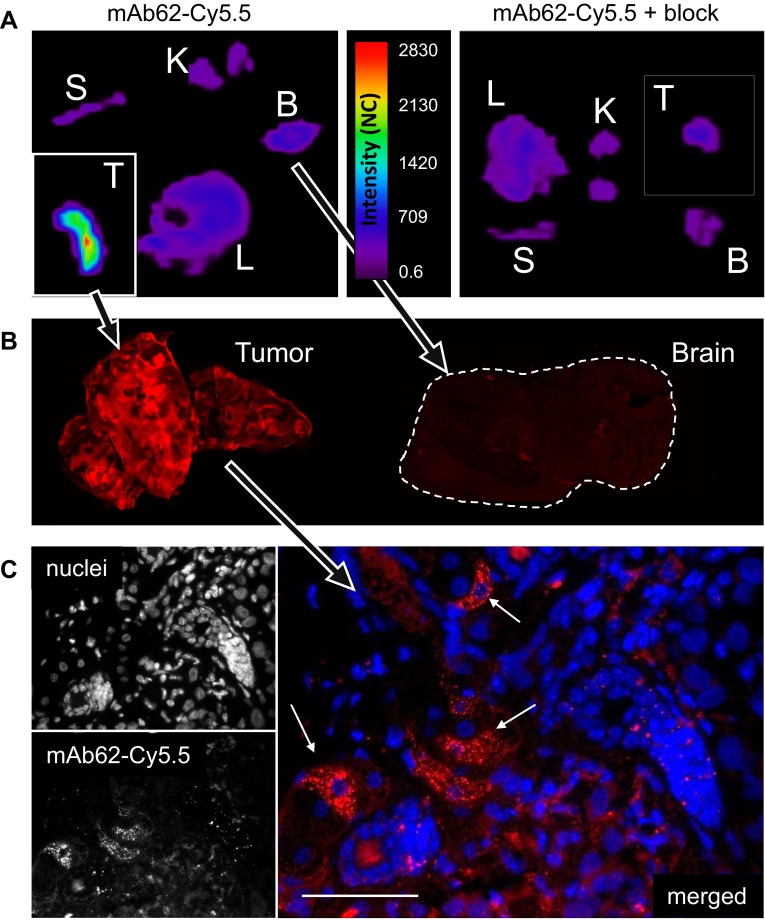
Ex vivo imaging of organs from MDA-MB-435S tumour-bearing mice after injection of mAb62-Cy5.5. **a** Tissue distribution of mAb62-Cy5.5 (25 µg; *left*) and mAb62-Cy5.5 co-injected with an excess of unlabelled mAb62 (50 µg; *right*) is shown (*T* tumour, *B* brain, *L* liver, *S* spleen, *K* kidney). Note that tumours (shown in *insets*) were scanned separately and are shown as 2 × larger images in comparison to the other organs. **b** Scans of slices of primary tumour and brain from mouse injected with 25 µg of mAb62-Cy5.5 performed with the Odyssey imaging system are presented. **c** NIRF microscopy of tumour slices confirmed the presence of mAb62-Cy5.5 (*red*) within tumour (*arrows*). Nuclei are stained with DAPI (*blue*). *Bar* 50 µm

Mice were killed and tumour as well as liver, spleen, kidney and brain was extracted and scanned ex vivo with the Optix MX2. As shown in Fig. 5a, ex vivo imaging confirmed that only the tumours of mice injected with mAb62-Cy5.5 showed strong fluorescence, but not tumours of the controls co-injected with the blocking antibody. No probe-derived fluorescence was observed in any other tested organs.

As K_v_10.1 is physiologically expressed in the brain, it was important to analyse whether mAb62-Cy5.5 is able to pass the blood-brain barrier and therefore detectable on brain tissue slices. Tumours and brains of mice injected with mAb62-Cy5.5 were thus embedded in paraffin and slices were scanned with the Odyssey imaging system (Fig. 5b). Strong fluorescence intensity was observed on the tumour slice, whereas no signal could be detected on the brain tissue slice. NIR fluorescence microscopy of tumour slices confirmed mAb62-Cy5.5-derived signals within tumour tissues (Fig. 5c).

### K_v_10.1-targeting antibody as a tool for delivery of a drug-activating enzyme to the tumour: proof of concept

Finally, in order to study the feasibility of the mAb62 to deliver diagnostic tools or therapeutics to the tumour, we conjugated the antibody with the drug-activating enzyme, β-D-galactosidase (mAb62-β-gal) and analysed its binding as well as measured the specific β-gal activity at the tumour cells in vitro and at the tumour site in vivo.

**Fig. 6:**
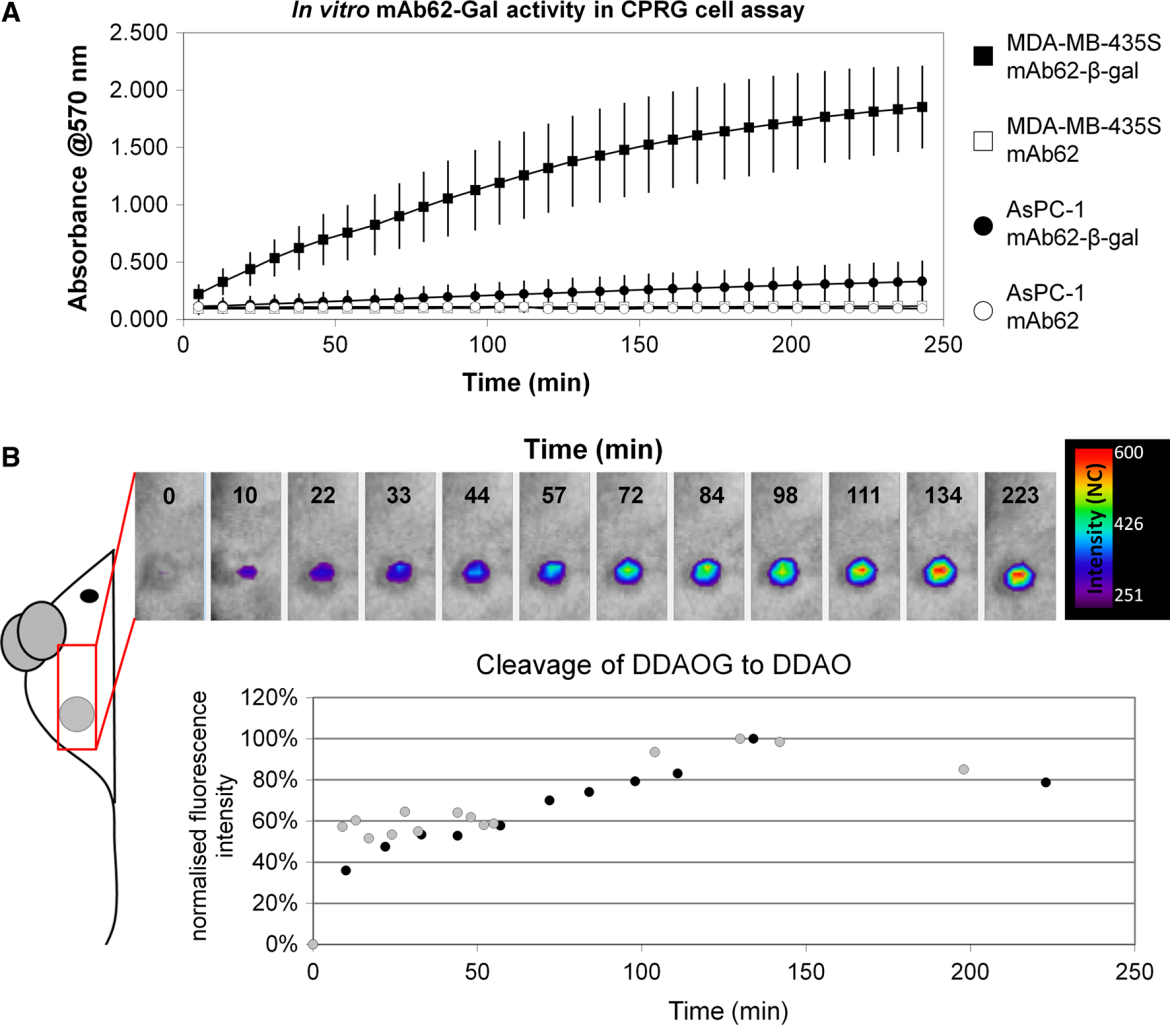
Binding and enzymatic activity of mAb62-β-gal in vitro and in vivo. **a** MDA-MB-435S and AsPC-1 cells were incubated with β-gal conjugated mAb62 (mAb62-β-gal) as well as with mAb62 as control. Cleavage of the β-gal substrate CPRG to chlorophenol red (570 nm absorbance) was measurable only in the MDA-MB-435S cells incubated with the conjugate, showing that mAb62-β-gal specifically binds to K_v_10.1-positive cells and that the enzyme is active in vitro. **b** mAb62-β-gal was applied to MDA-MB-435S tumour-bearing mice (*n* = 2) and allowed to bind to the tumour for 24 h; 5 mg of DDAOG was administered i.v. and cleavage to DDAO resulting in an excitation shift from 465 to 646 nm was imaged with Optix MX2. Fluorescence intensity (NC) of the DDAO measured over the tumour increased within the first 3 h after application of DDAOG, confirming that mAb62-β-gal binds to the tumour and the enzyme remains active in vivo

To this end, K_v_10.1-expressing MDA-MB-435S and control AsPC-1 cells were incubated for 30 min in vitro with mAb62-β-gal as well as with the non-conjugated mAb62 as a negative control. After careful washing of the unbound conjugate, cleavage of the β-gal substrate CPRG to chlorophenol red (570 nm absorbance) by the bound β-gal conjugate was measured. A positive reaction was only detected on the MDA-MB-435S cells incubated with the mAb62-β-gal construct (Fig. 6a). The control AsPC-1 cells incubated with the mAb62-β-gal showed only a very low cleavage of CPRG to chlorophenol red. As expected, β-gal activity was not measurable on the cells incubated with the unconjugated mAb62.

Finally, the in vivo activity of mAb62-β-gal was measured in MDA-MB-435S tumour-bearing mice (*n* = 2) using the β-gal substrate DDAOG, which shifts its excitation maximum from 465 to 646 nm upon cleavage to DDAO. In the first step, mAb62-β-gal was applied to MDA-MB-435S tumour-bearing mice and allowed to bind to the tumour for 24 h, the time when the mAb62-Cy5.5 achieved the highest tumour accumulation. Subsequently, we administered DDAOG i.v. and measured the fluorescence intensity of the DDAO over the tumour area over ~3.5 h. As shown in Fig. 6b, fluorescence intensity increased within the first 2 h after application, confirming that mAb62-β-gal binds to the tumour and the enzyme galactosidase remains active in vivo at the tumour site.

## Discussion

Along with their role as critical mediators of numerous physiological processes, ion channels are increasingly in the focus of oncology. Due to their role in the regulation of various steps of neoplastic progression they have been suggested as promising tools for cancer treatment and diagnosis.

Here we present a systematic characterisation of the monoclonal antibody mAb62, targeting the K_v_10.1 potassium channel in vivo in a nude mouse subcutaneous tumour model as a potential tool for cancer targeting. This particular antibody specifically recognises the full-length K_v_10.1, but not its shorter splice variants, which do not display the corresponding epitope (Ramos Gomes et al. 2015). By NIRF imaging we showed a selective binding of the NIR fluorescently labelled mAb62 to K_v_10.1-expressing MDA-MB-435S tumours in vivo, with a peak at 2 days post injection, which could be blocked by an excess of unlabelled mAb62. Furthermore, we show that the antibody is detectable at the tumour site for up to 2 weeks in vivo. Comparable binding of antibodies up to several days post injection and with maximum intensities between 1 and 2 days has previously been shown by in vivo fluorescence imaging (Napp et al. 2010). By contrast, smaller molecules, such as RGD peptides, usually show a maximum fluorescence intensity as early as a few hours post injection, mainly due to more rapid renal clearance (Mathejczyk et al. 2011).

We have chosen NIRF imaging to assess the binding and biodistribution of mAb62 as this technique utilises non-ionising radiation and therefore has the important advantage of being harmless, in contrast to other functional imaging modalities such as PET or SPECT. In addition, it is a simple, quick and relatively cheap method, which delivers a variety of information such as on target expression, probe biodistribution and tissue characteristics such as pH and oxygen levels or enzymatic activity (Napp et al. 2010, 2011; Mathejczyk et al. 2011, 2012). Moreover, the use of NIR light allows for relatively deep tissue penetration of up to 2 cm, which means that even deeply located structures, such as a fluorescently labelled pancreatic tumour, can be easily detected within a living mouse (Napp et al. 2010).

Since we applied a subcutaneous tumour mouse model, in which tumours are visible with the naked eye, the detected fluorescence could be clearly located to the tumour site. In contrast, orthotopically transplanted tumours, especially those located deep within the body, as for example pancreatic cancer, are often much more difficult to correlate to anatomical structures. In such cases NIRF imaging data have to be overlaid with data obtained from an anatomical imaging modality such as CT, as already shown for orthotopic pancreatic or mammary tumours (Dullin et al. 2009).

We confirmed the specificity of the fluorescence signals by showing that the fluorescence detected over the tumour 24 h after injection of mAb62-Cy5.5 had almost the same lifetime (1.6 ns) as the one of the mAb62-Cy5.5 measured in vitro (1.5 ns). The fact that the same lifetime was also measured all over the body, except the gastrointestinal tract, is most likely due to the probe still present in the circulation and not yet completely cleared from the blood. This is not surprising as whole antibodies such as the mAb62 are known to have relatively long circulation times (Wang et al. 2008). The long fluorescence lifetime of 1.9–2.3 ns over the gastrointestinal tract, which was also observed in the pre-contrast scans, was already described in our previous studies and can be explained by the autofluorescence of digested food despite feeding the mice with chlorophyll-depleted pellets (Napp et al. 2010).

The most likely source of harmful effects from tools targeting ion channels is the direct influence they could have on the physiology of excitable tissue. As K_v_10.1 is physiologically expressed in the central nervous system, and because previous immunohistochemistry experiments (Hemmerlein et al. 2006) showed specific binding of mAb62 to rat brain slices, it was of extreme importance to exclude the diffusion and binding of the i.v. applied mAb62 to the brain tissue. Using in vivo fluorescence imaging as well as ex vivo imaging of the excised organs and tissue slices we could show that mAb62-Cy.5.5 did not accumulate within brain tissue. The slight increase of the fluorescence intensity over the cranial area in in vivo dorsal scans performed 24 h after probe injection was comparable to the overall increase of the whole-body fluorescence. This confirms that the signals measured over the brain were most probably due to the circulating probe rather than to the specific accumulation of mAb62-Cy.5.5 in the brain. In general, whole antibodies show a relatively low distribution to the brain, with brain-to-plasma ratios as low as 1:500, mainly due to the blood brain barrier and rapid turnover of brain interstitial fluids. This makes whole antibodies often more suitable for tumour targeting than smaller ligands, especially when brain targeting is not desired (Wang et al. 2008). Monoclonal antibodies are increasingly applied in clinical practice, e.g., for treatment of cancer, transplant rejection or infectious diseases (Catapano and Papadopoulos 2013). In general, their off-target toxicity is relatively low and mostly restricted to hypersensitivity reactions and immunogenicity, which can be easily reduced by production of genetically engineered chimeric (mouse-human) or humanised monoclonal antibodies.

**Fig. 7:**
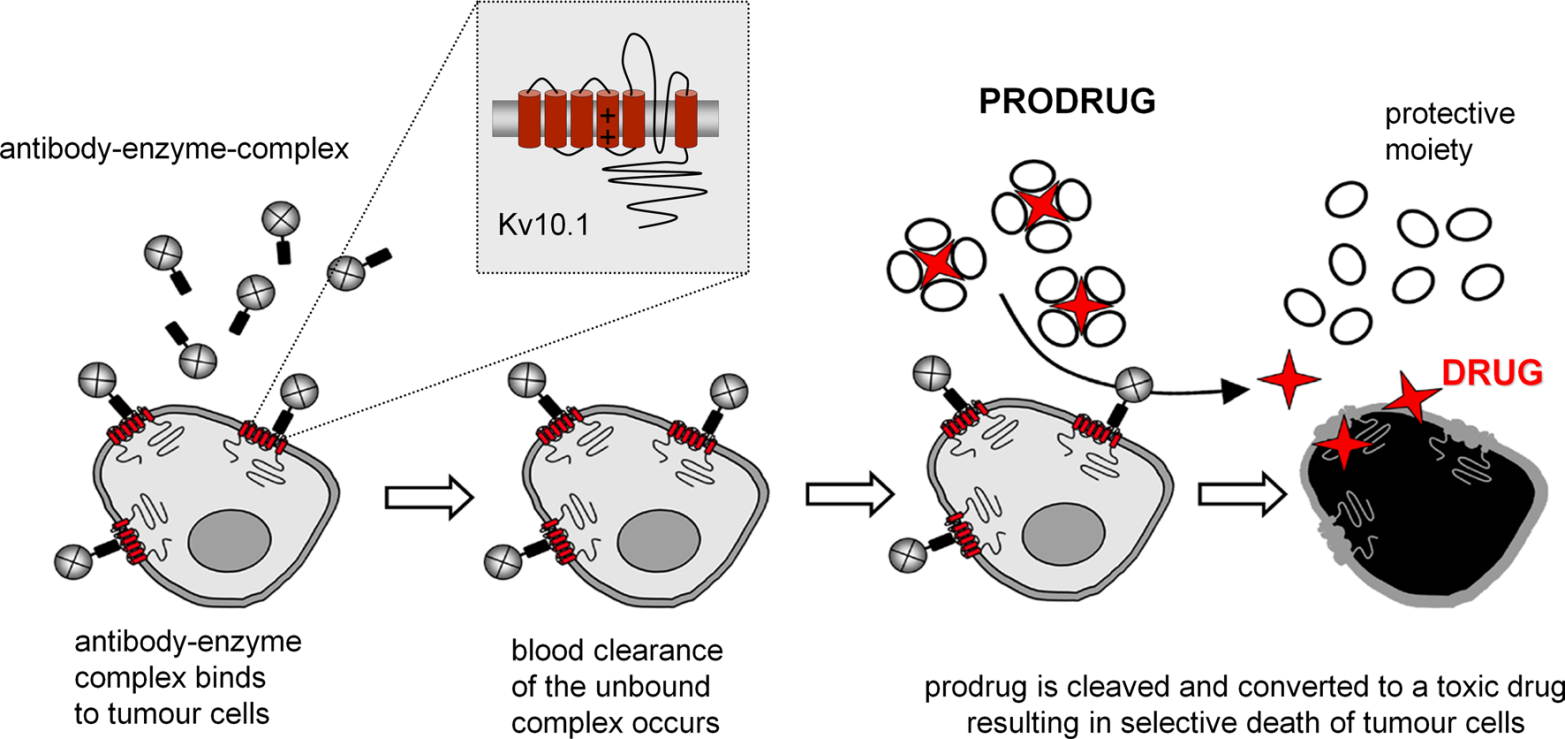
Schematic representation of K_v_10.1-based antibody-directed enzyme prodrug therapy (ADEPT). Antibody targeting K_v_10.1 can be used to carry a drug-activating enzyme such as β-D-galactosidase (β-gal) to the tumour. Specific binding of the antibody-enzyme complex at the tumour site results in spatially restricted cleavage of the nontoxic prodrug to an active anticancer drug

In a proof-of-concept experiment we showed that the mAb62-β-gal construct can specifically deliver drug-activating enzyme to the tumour. We confirmed that the mAb62-β-gal conjugate not only specifically binds to K_v_10.1-expressing cells in vitro and accumulates within K_v_10.1-expressing tumours, but also retains its enzymatic activity on tumour cells, both in vitro and in vivo. Therefore, mAb62 conjugated to the enzyme β-D-galactosidase could represent a tool for tumour-specific therapies such as the antibody-directed enzyme prodrug therapy (ADEPT; Fig. 7) (Alves et al. 2009) in which a tumour-targeting ligand carries a drug-activating enzyme to the tumour tissue and a subsequently administered non-toxic prodrug is locally converted to a cytotoxic drug by the tumour-bound enzyme.

Here we show the use of fluorescence imaging to evaluate the specificity of the antibody binding or the activity of the antibody-enzyme conjugate at the tumour site. Additional anatomical imaging, such as CT, could be considered for anatomical correlation as has been previously shown (Alves et al. 2009). Alternatively, bioluminescence imaging could be applied upon transplantation of genetically modified tumour cells to monitor therapeutic efficacy by assessing tumour progression in vivo over time.

Antibodies and small molecule compounds targeting K_v_10.1 or blocking K_v_10.1 channel function have already been proposed for therapeutic purposes (Pardo et al. 2012). A single chain antibody scFv62, derived from mAb62, was fused to the tumour necrosis factor (TNF)-related apoptosis-inducing ligand (TRAIL) and was shown to induce apoptosis and to sensitise prostate cancer cells to chemotherapeutics in vitro (Hartung et al. 2011; Hartung and Pardo 2016). Another monoclonal antibody, functioning as a channel blocker and able to inhibit K_v_10.1 current, was shown to not only reduce the growth of cancer cells in vitro, but also tumour progression in vivo in subcutaneous MDA-MB-435S xenografts and in human pancreatic cancer explants (Gomez-Varela et al. 2007). Similarly, astemizole, a small-molecule open channel blocker of K_v_10.1, was reported to inhibit growth of K_v_10.1-expressing cancer cells in vitro and in vivo (Downie et al. 2008; Garcia-Quiroz et al. 2014; Chavez-Lopez et al. 2015).

Both, monoclonal antibodies and small molecules are promising tools for cancer targeting, but the individual advantages and disadvantages of each have to be taken into consideration (Imai and Takaoka 2006). A major impediment of monoclonal antibodies is that they are typically produced in mice and therefore commonly induce an immune response in humans known as a human anti-mouse antibody (HAMA). This limits their repeated use in most patients, though this problem can currently be solved relatively easily by using humanised antibodies. The large size of whole antibodies (~150 kDa) results in prolonged blood half-lives and slow hepatic clearance, whereas the many times smaller molecules are usually cleared much faster and via renal excretion. For example, the half-life of cetuximab, a chimeric monoclonal antibody targeting the epithelial growth factor receptor (EGFR) and clinically approved for treatment of several cancer types, is between 3.1 and 7.8 days in humans, whereas the half-life of a small molecule drug targeting the same molecule EGFR is only 36 h, requiring a different dosing scheme (Imai and Takaoka 2006). This frequently influences the administration route: while monoclonal antibodies are usually injected intravenously, small molecules are often administered orally. Furthermore, and probably most importantly, cell membranes are generally impermeable to IgGs whereas many small molecules can easily pass into the cell. This means that whole antibodies most of the time act on cell surface targets, such as the K_v_10.1, while small molecule drugs more often target intracellular molecules.

**Fig. 8:**
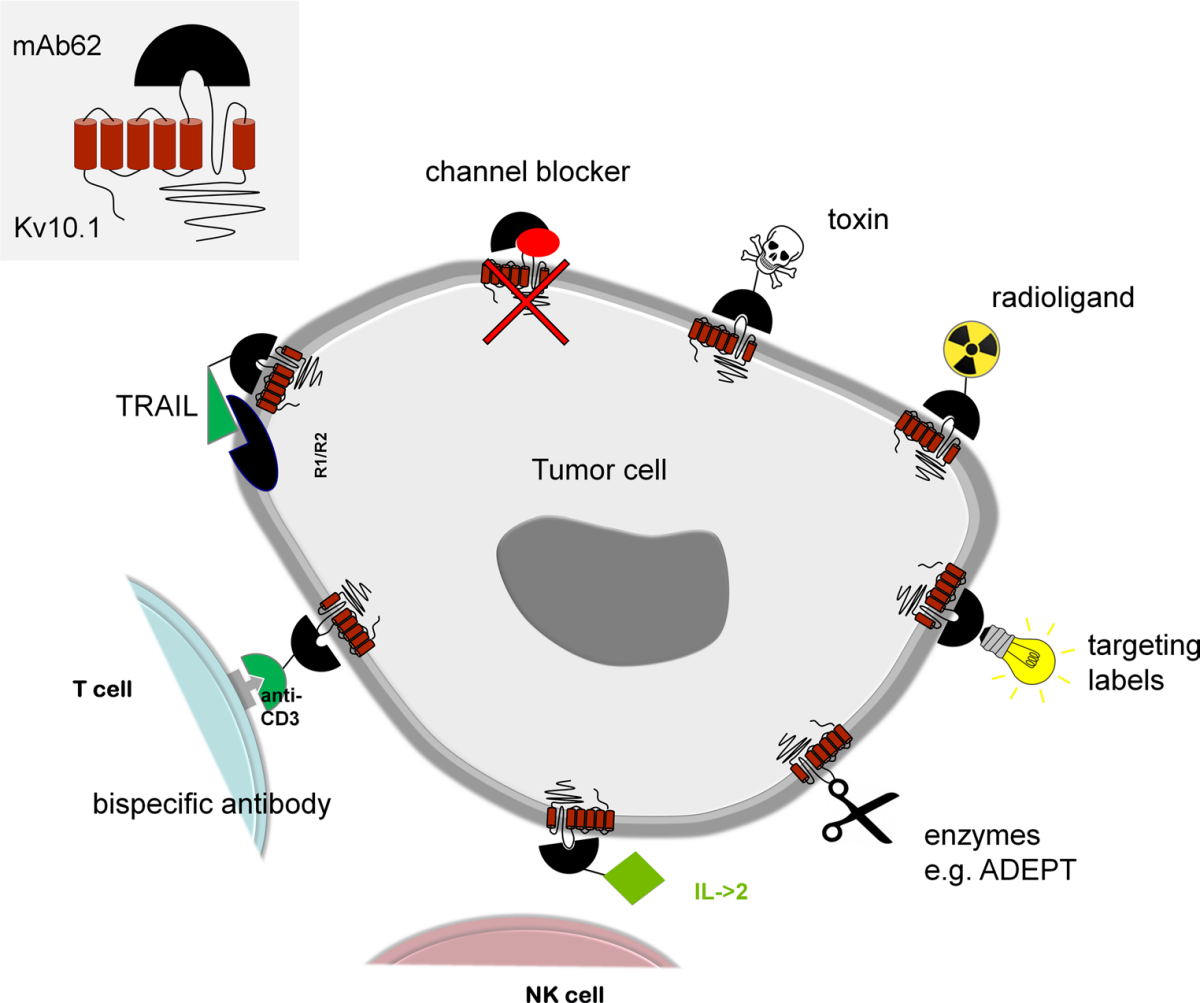
Potential therapies based on targeting of the K_v_10.1 channel. Potential therapies targeting K_v_10.1 may include use of channel blockers, toxins, radioligands, enzymes or apoptosis-inducing ligands such as TRAIL. They might also be used for selective activation of the host immune system, for example using bispecific antibodies targeting CD3 or cytokines such as IL-2, but also for labelling of tumour cells, e.g. with fluorescent dyes

Ion channels are especially interesting targets for therapeutic interventions as they are easily accessible from the extracellular milieu. The monoclonal antibody used in our study may not only be a valuable tool for ADEPT, but can also be used for the targeted delivery of toxic compounds to the tumours, like radioligands or bacterial toxins (Fig. 8). These so-called immunotoxins are already used in clinical trials and some show promise for further clinical evaluation. The concept of fusing antibodies to apoptosis-inducing ligands (e.g., TRAIL) has the advantage of low immunogenicity, because the ligands are a part of the immune system and, unlike toxins, will not be detected as foreign substances. An alternative approach is the selective activation of the host immune system and initiation of an immune response in the tumour. For this purpose, bispecific antibodies can be used that simultaneously target a tumour-specific antigen and the CD3 receptor to transport and activate cytotoxic T cells to and at the tumour site. Also antibody-cytokine fusion proteins enhance the direct antitumour effect of the antibody and concentrate the cytokine (e.g. IL-2) in the tumour microenvironment without causing severe toxic side effects of systemic high-dose cytokine administration. A combination of different antibody constructs may be the right course for an effective therapeutic strategy in the future.

## References

[CR1] Alves F, Dullin C, Napp J, Missbach-Guentner J, Jannasch K, Mathejczyk J, Pardo LA, Stuhmer W, Tietze LF (2009). Concept of a selective tumour therapy and its evaluation by near-infrared fluorescence imaging and flat-panel volume computed tomography in mice. Eur J Radiol.

[CR2] Arcangeli A, Becchetti A (2015). Novel perspectives in cancer therapy: targeting ion channels. Drug Resist Updat.

[CR3] Catapano AL, Papadopoulos N (2013). The safety of therapeutic monoclonal antibodies: implications for cardiovascular disease and targeting the PCSK9 pathway. Atherosclerosis.

[CR4] Chavez-Lopez MD, Perez-Carreon JI, Zuniga-Garcia V, Diaz-Chavez J, Herrera LA, Caro-Sanchez CH, Acuna-Macias I, Gariglio P, Hernandez-Gallegos E, Chiliquinga AJ, Camacho J (2015). Astemizole-based anticancer therapy for hepatocellular carcinoma (HCC), and Eag1 channels as potential early-stage markers of HCC. Tumor Biol.

[CR5] Collins TJ (2007). ImageJ for microscopy. Biotechniques.

[CR6] Ding XW, Luo HS, Jin X, Yan JJ, Ai YW (2007). Aberrant expression of Eag1 potassium channels in gastric cancer patients and cell lines. Med Oncol.

[CR7] Ding XW, Yan JJ, An P, Lu P, Luo HS (2007). Aberrant expression of ether a go-go potassium channel in colorectal cancer patients and cell lines. World J Gastroenterol.

[CR8] Downie BR, Sanchez A, Knotgen H, Contreras-Jurado C, Gymnopoulos M, Weber C, Stuhmer W, Pardo LA (2008). Eag1 expression interferes with hypoxia homeostasis and induces angiogenesis in tumors. J Biol Chem.

[CR9] Dullin C, Zientkowska M, Napp J, Missbach-Guentner J, Krell HW, Muller F, Grabbe E, Tietze LF, Alves F (2009). Semiautomatic landmark-based two-dimensional-three-dimensional image fusion in living mice: correlation of near-infrared fluorescence imaging of Cy5.5-labeled antibodies with flat-panel volume computed tomography. Mol Imaging.

[CR10] Garcia-Quiroz J, Garcia-Becerra R, Santos-Martinez N, Barrera D, Ordaz-Rosado D, Avila E, Halhali A, Villanueva O, Ibarra-Sanchez MJ, Esparza-Lopez J, Gamboa-Dominguez A, Camacho J, Larrea F, Diaz L (2014). In vivo dual targeting of the oncogenic Ether-a-go-go-1 potassium channel by calcitriol and astemizole results in enhanced antineoplastic effects in breast tumors. BMC Cancer.

[CR11] Gomez-Varela D, Zwick-Wallasch E, Knotgen H, Sanchez A, Hettmann T, Ossipov D, Weseloh R, Contreras-Jurado C, Rothe M, Stuhmer W, Pardo LA (2007). Monoclonal antibody blockade of the human Eag1 potassium channel function exerts antitumor activity. Cancer Res.

[CR12] Hammer Ø, Harper D, Ryan P (2001). PAST-palaeontological statistics, ver. 1.89. Palaeontol Electron.

[CR13] Hartung F, Pardo LA (2016) Guiding TRAIL to cancer cells through Kv10.1 potassium channel overcomes resistance to doxorubicin. Eur Biophys J. doi:10.1007/s00249-016-1149-7 10.1007/s00249-016-1149-7 PMC5045482 27350552

[CR14] Hartung F, Stuhmer W, Pardo LA (2011). Tumor cell-selective apoptosis induction through targeting of K_V_10.1 via bifunctional TRAIL antibody. Mol Cancer.

[CR15] Hemmerlein B, Weseloh RM, Mello de Queiroz F, Knotgen H, Sanchez A, Rubio ME, Martin S, Schliephacke T, Jenke M, Heinz Joachim R, Stuhmer W, Pardo LA (2006). Overexpression of Eag1 potassium channels in clinical tumours. Mol Cancer.

[CR16] Imai K, Takaoka A (2006). Comparing antibody and small-molecule therapies for cancer. Nat Rev Cancer.

[CR17] Lang F, Stournaras C (2014). Ion channels in cancer: future perspectives and clinical potential. Philos Trans R Soc Lond B Biol Sci.

[CR18] Martinez R, Stuhmer W, Martin S, Schell J, Reichmann A, Rohde V, Pardo L (2015). Analysis of the expression of Kv10.1 potassium channel in patients with brain metastases and glioblastoma multiforme: impact on survival. BMC Cancer.

[CR19] Mathejczyk JE, Pauli J, Dullin C, Napp J, Tietze LF, Kessler H, Resch-Genger U, Alves F (2011). Spectroscopically well-characterized RGD optical probe as a prerequisite for lifetime-gated tumor imaging. Mol Imaging.

[CR20] Mathejczyk JE, Pauli J, Dullin C, Resch-Genger U, Alves F, Napp J (2012). High-sensitivity detection of breast tumors in vivo by use of a pH-sensitive near-infrared fluorescence probe. J Biomed Opt.

[CR21] Mello de Queiroz F, Suarez-Kurtz G, Stuhmer W, Pardo LA (2006). Ether a go-go potassium channel expression in soft tissue sarcoma patients. Mol Cancer.

[CR22] Napp J, Dullin C, Muller F, Uhland K, Petri JB, van de Locht A, Steinmetzer T, Alves F (2010). Time-domain in vivo near infrared fluorescence imaging for evaluation of matriptase as a potential target for the development of novel, inhibitor-based tumor therapies. Int J Cancer.

[CR23] Napp J, Behnke T, Fischer L, Wurth C, Wottawa M, Katschinski DM, Alves F, Resch-Genger U, Schaferling M (2011). Targeted luminescent near-infrared polymer-nanoprobes for in vivo imaging of tumor hypoxia. Anal Chem.

[CR24] Pardo LA, Stuhmer W (2014). The roles of K(+) channels in cancer. Nat Rev Cancer.

[CR25] Pardo LA, del Camino D, Sanchez A, Alves F, Bruggemann A, Beckh S, Stuhmer W (1999). Oncogenic potential of EAG K(+) channels. EMBO J.

[CR26] Pardo LA, Gomez-Varela D, Major F, Sansuk K, Leurs R, Downie BR, Tietze LF, Stuhmer W (2012). Approaches targeting K_V_10.1 open a novel window for cancer diagnosis and therapy. Curr Med Chem.

[CR27] Ramos Gomes F, Romaniello V, Sanchez A, Weber C, Narayanan P, Psol M, Pardo LA (2015). Alternatively spliced isoforms of K_V_10.1 potassium channels modulate channel properties and can activate cyclin-dependent kinase in xenopus oocytes. J Biol Chem.

[CR28] Tung CH, Zeng Q, Shah K, Kim DE, Schellingerhout D, Weissleder R (2004). In vivo imaging of beta-galactosidase activity using far red fluorescent switch. Cancer Res.

[CR29] Wang W, Wang EQ, Balthasar JP (2008). Monoclonal antibody pharmacokinetics and pharmacodynamics. Clin Pharmacol Ther.

[CR30] Weber C, de Queiroz FM, Downie BR, Suckow A, Stuhmer W, Pardo LA (2006). Silencing the activity and proliferative properties of the human EagI potassium channel by RNA interference. J Biol Chem.

